# Twelve years' detection of respiratory viruses by immunofluorescence in hospitalised children: impact of the introduction of a new respiratory picornavirus assay

**DOI:** 10.1186/1471-2334-11-41

**Published:** 2011-02-07

**Authors:** Christine D Sadeghi, Christoph Aebi, Meri Gorgievski-Hrisoho, Kathrin Mühlemann, Maria Teresa Barbani

**Affiliations:** 1 Institute for Infectious Diseases, University of Bern, Friedbühlstrasse 51, CH-3010 Bern, Switzerland; 2 University Children's Hospital, Inselspital, Freiburgstrasse, CH- 3010 Bern, Switzerland

## Abstract

**Background:**

Direct immunofluorescence assays (DFA) are a rapid and inexpensive method for the detection of respiratory viruses and may therefore be used for surveillance. Few epidemiological studies have been published based solely on DFA and none included respiratory picornaviruses and human metapneumovirus (hMPV). We wished to evaluate the use of DFA for epidemiological studies with a long-term observation of respiratory viruses that includes both respiratory picornaviruses and hMPV.

**Methods:**

Since 1998 all children hospitalized with respiratory illness at the University Hospital Bern have been screened with DFA for common respiratory viruses including adenovirus, respiratory syncytial virus (RSV), influenza A and B, and parainfluenza virus 1-3. In 2006 assays for respiratory picornaviruses and hMPV were added. Here we describe the epidemiological pattern for these respiratory viruses detected by DFA in 10'629 nasopharyngeal aspirates collected from 8'285 patients during a 12-year period (1998-2010).

**Results:**

Addition of assays for respiratory picornaviruses and hMPV raised the proportion of positive DFA results from 35% to 58% (p < 0.0001). Respiratory picornaviruses were the most common viruses detected among patients ≥1 year old. The seasonal patterns and age distribution for the studied viruses agreed well with those reported in the literature. In 2010, an hMPV epidemic of unexpected size was observed.

**Conclusions:**

DFA is a valid, rapid, flexible and inexpensive method. The addition of assays for respiratory picornaviruses and hMPV broadens its range of viral detection. DFA is, even in the "PCR era", a particularly adapted method for the long term surveillance of respiratory viruses in a pediatric population.

## Background

Respiratory infections are a major cause of morbidity and hospitalizations in children [1], of which a significant proportion are caused by viruses [2,3]. Surveillance of respiratory viruses is important to predict seasonal epidemics, to define patient risk groups and to allocate hospital resources, as well as to describe the burden and characteristics of emerging viruses [4].

In the field of virology, the most commonly used diagnostic methods for virus detection are culture, rapid culture (such as shell vial assay), direct immunofluorescence staining of clinical specimens (DFA), and PCR. PCR is attractive due to its high sensitivity and broad range of virus detection. PCR-based studies have suggested the important role of respiratory picornaviruses (rhinovirus and enterovirus) as a leading cause of lower respiratory tract infections in children [5], in particular wheezing illnesses such as bronchiolitis [6,7], wheezy bronchitis [8] and asthma exacerbations [9], but also pneumonia [2]. In addition, PCR has allowed for the detection of new respiratory viruses, such as hMPV [10], which has been implicated in upper and lower respiratory tract infections in children [11-13]. It is widely believed that nowadays, epidemiological studies on respiratory viruses can only be done with PCR.

However, the high sensitivity of PCR is also a limitation of the technique. A significant proportion of asymptomatic children test positive by PCR to respiratory viruses [14-16], and picornavirus RNA can be detected by PCR up to 5 weeks after an acute infection [17]. Therefore, epidemiological studies based on PCR may overestimate the burden of certain viruses, in particular the common respiratory picornaviruses.

DFA has lower sensitivity than PCR, but this may be an advantage for the detection of clinically relevant infections [18,19]. Moreover, DFA is more rapid and less expensive than PCR and can therefore be used for real-time, routine surveillance of respiratory viruses, which would be difficult by PCR because of the high costs [20,21]. Nevertheless only few epidemiological studies have been published based solely on DFA [22,23], and none of them included testing for respiratory picornaviruses or hMPV, because antibodies for the detection of hMPV have only recently become available and there are no commercial antibodies for the detection of respiratory picornaviruses. Recently, our group reported the validity of immunofluorescence for the detection of picornaviruses directly in respiratory samples using monoclonal antibodies originally designed for the identification of enterovirus in culture [24].

We aimed to evaluate the use of DFA for epidemiological studies of respiratory viruses, now that assays for respiratory picornaviruses and hMPV are available. We performed a retrospective analysis among pediatric patients hospitalized with respiratory tract infections between 1998 and 2010 at the University Hospital Bern. Prospective DFA testing in nasopharyngeal aspirates has been used routinely in this institution for adenovirus (ADV), respiratory syncytial virus (RSV), influenza A and B (IFA and IFB), and parainfluenza 1-3 (PIV 1-3) since 1998, and additionally for hMPV and respiratory picornaviruses since 2006.

## Methods

### Patient population and sample collection

The study was approved by the Ethics Committee of the University Hospital of Bern in accordance with cantonal ethical regulations (Nr. E 10-01-10). The study included consecutive respiratory tract samples from children under the age of 17 years, who were hospitalized at the Department of Pediatrics, University Hospital Bern, between May 1^st ^1998 and April 30^th ^2010. During the entire study period the pediatrics department had the policy of screening children for respiratory viruses if they were hospitalized with a respiratory illness or if they developed respiratory symptoms during their hospital stay.

A total of 12'189 respiratory samples were collected. After exclusion criteria, 10'629 samples remained for the retrospective analysis of DFA results. The exclusion criteria were as follows: samples other than nasopharyngeal aspirates, samples containing less than 20 epithelial cells and samples not tested against the whole viral panel; results of samples from the same patient taken within a time period of 7 days (considered part of the same respiratory episode); results for the month of August 2009, since during this time period practically all respiratory samples were tested by PCR rather than DFA due to the influenza A H1N1 pandemic.

### Direct immunofluorescence testing (DFA) for respiratory viruses

All samples were analysed at the Institute for Infectious Diseases, University of Bern. The methods used have previously been described [24].

Between May 1^st ^1998 and August 31^st ^2007, the Light Diagnostics Respiratory Viral Screen DFA (Chemicon International, now Millipore) and single fluorescein-conjugated monoclonal antibodies against ADV, RSV, IFA, IFB and PIV 1-3 were used. From September 1^st ^2007 to April 30^th ^2010, the D3 Ultra DFA Respiratory Virus Screening & ID Kit (Diagnostic Hybrids) was used for the same viruses. Starting in March 2006, the Light Diagnostics Pan-Enterovirus Reagent "Blend" (Chemicon International/Millipore) was introduced for the detection of respiratory picornaviruses. This assay is formally an indirect immunofluorescence assay, as described elsewhere [24], and does not allow the differentiation between rhinoviruses and enteroviruses. In November 2006, the DFA Metapneumovirus Identification Kit (Diagnostic Hybrids) was added to the screening.

### Statistical analysis

An epidemiological year was defined as May 1^st ^to April 30^th ^of the following year. Summer was defined as the months July to September, and winter as January to March. Epidemiological years were designated "odd" if the month of January was in an odd year, and they were labelled "even" if the month of January was in an even year.

All statistical analyses were performed with the GraphPad Prism 5^® ^software tool (GraphPad Software, Inc.). Proportions were compared using the chi-square test. Medians were compared with the Kruskal-Wallis test and Dunn's multiple comparison test. A cut-off of p ≤ 0.05, two tailed, was used for all statistical analyses.

## Results

### Population

A total of 10'629 samples from 8'285 patients were analysed. The median age of the study population was 11 months (range 0-17 years) and 57.5% were boys.

### Rate of viral detection

**Figure 1:**
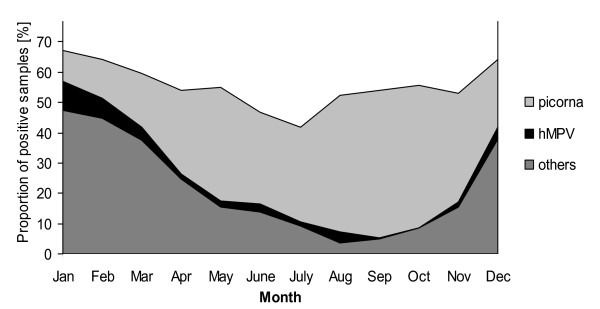
**Mean monthly distribution of respiratory viruses detected by DFA in nasopharyngeal aspirates from hospitalized children from November 2006 to April 2010**. picorna = respiratory picornaviruses; hMPV = human metapneumovirus; others = adenovirus, respiratory syncytial virus, influenza A and B, parainfluenza viruses 1-3.

Before the addition of DFA for picornavirus and hMPV, the rate of viral detection was dominated by the RSV season, with a yearly average rate of 35%, peaks of up to 64% (average 46%) in winter seasons, and troughs as low as 4% (average 16%) during summer time. The addition of DFA for picornaviruses increased the positivity rate, and dampened the seasonal variations. The positivity rate after 2006 was on average 65% in winter and 52% in summer, with a yearly average of 58% (versus 35%; p < 0.0001) (Figure 1, Table 1). For comparison, analysis performed on 256 specimens in parallel to DFA screening with the xTag Respiratory Viral Panel (Luminex Molecular Diagnostics) between November 2006 and September 2007 yielded a positivity rate of 78%. The higher detection rate by PCR could mostly be attributed to increased detection of respiratory picornaviruses; 57 from the 78 additional positive results were respiratory picornaviruses (unpublished data).

**Table 1:** Proportion (%) of positive DFA results in nasopharyngeal aspirates by virus and by year, 1998-2010

Epidemiogical years	98/99	99/00	00/01	01/02	02/03	03/04	04/05	05/06	06/07	07/08	08/09	09/10	1998- 2010	1998- 2006	2006- 2010
**ADV**	3	3	3	3	1	2	4	4	3	2	2	3	**3**		
**RSV**	32	15	32	12	31	15	26	13	26	15	23	12	**21**		
**IFA**	7	12	2	6	4	6	6	0.2	3	2	3	2	**4**		
**IFB**	2	0	0	2	1	0	0.1	2	0	0.2	0.4	0	**0.6**		
**PIV**	5	4	5	3	4	6	3	2	2	3	4	4	**4**		
**hMPV**									0.2	4	1	8	**3**		
**Picorna**								2	24	26	33	28	**27**		

**Total**	49	34	43	26	41	29	38	24	57	53	66	57	**44**	**35**	**58**
**Summer**	28	16	15	10	24	20	8	**4**	48	48	63	43		**16**	**52**
**Winter**	58	42	58	34	41	40	**64**	33	60	60	70	68		**46**	**65**

An epidemiological year lasts from May 1st to April 30th of the following year

ADV = adenovirus; RSV = respiratory syncytial virus; IFA = influenza A; IFB = influenza B; PIV = parainfluenza viruses 1-3;

hMPV = human metapneumovirus; picorna = respiratory picornaviruses

### Pathogens

**Figure 2:**
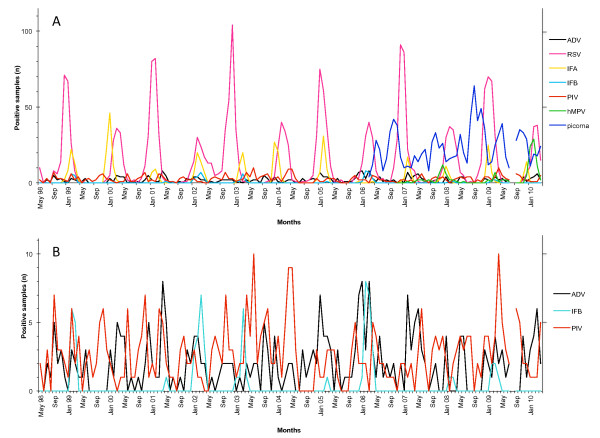
**Monthly distribution of respiratory viruses detected by DFA in nasopharyngeal aspirates from hospitalized children between May 1998 and April 2010, with introduction of the hMPV and respiratory picornavirus assays in 2006**. A: All viruses detected. B: Detailed view of ADV, IFB and PIV (note that the scale is different than in A). ADV = adenovirus; RSV = respiratory syncytial virus; IFA = influenza A; IFB = influenza B; PIV = parainfluenza viruses 1-3; hMPV = human metapneumovirus; picorna = respiratory picornaviruses.

Respiratory picornaviruses were the most common pathogens detected overall in our study population after the introduction of the assay (**27% **versus 21% for RSV; p < 0.0001) (Table 1). They were present year round, with peaks in the spring and the fall. During the summer time, respiratory picornaviruses also accounted for the majority of viral respiratory infections (Figure 1). Low prevalence of respiratory picornaviruses during winter time coincided with the winter peaks caused by RSV, influenza, or hMPV, and this was the only time during the year when respiratory picornaviruses were not the most commonly detected respiratory virus (Figure 2).

RSV was the second most commonly detected pathogen after picornaviruses (overall prevalence of 21%), but the most prevalent virus during the winter months (Table 1, Figure 2). It manifested a biennial pattern, with large winter seasons in odd years alternating with smaller ones in even years.

Influenza A virus was found in 4.1% of all samples and caused yearly winter epidemics, except during the winter season 2005-2006 when it was replaced by influenza B virus (Table 1, Figure 2).

**Figure 3:**
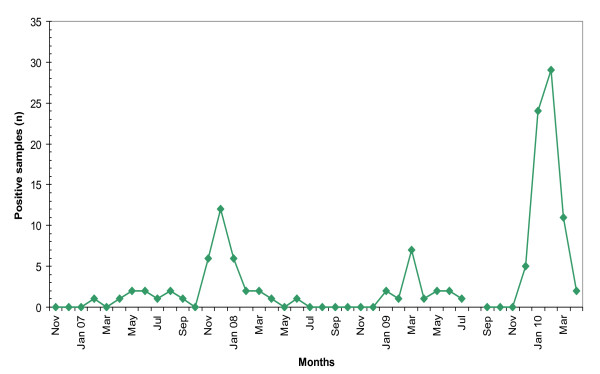
**Monthly human metapneumovirus detection by DFA in nasopharyngeal aspirates from hospitalized children between November 2006 and April 2010**.

hMPV was detected in 3.4% of all samples collected after November 2006 (Table 1). Yearly hMPV activity varied from being almost absent during the winter season 2006 to 2007, to causing yearly winter outbreaks during the following years (Figure 2, Figure 3). In early 2010, viral activity surpassed previous years, with more cases observed within 5 months (71 cases between December 2009 and April 2010) than during the entire previous period since introduction of the DFA test (56 cases between November 2006 and November 2009).

### Age distribution

**Figure 4:**
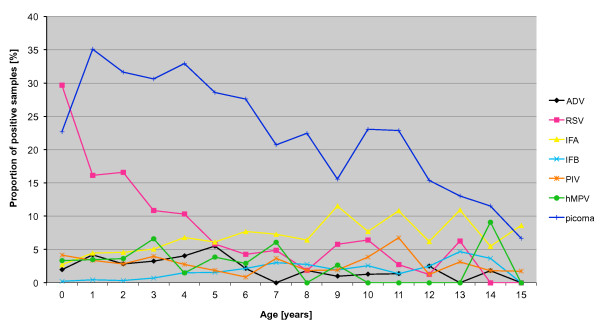
**Proportion of nasopharyngeal aspirates from hospitalized children positive by DFA for respiratory viruses according to age and virus**. Based on 1998-2010 data for adenovirus (ADV), respiratory syncytial virus (RSV), influenza A (IFA), influenza B (IFB) and parainfluenza viruses 1-3 (PIV). Based on 2006-2010 data for human metapneumovirus (hMPV) and respiratory picornaviruses (picorna). Note: due to a low number of samples, data for the 16-17 year olds is not shown.

We compared the proportion of samples positive for a given virus by age (Figure 4). Respiratory picornaviruses were the most common pathogens in children ≥1 years (**1-4 years**: 33% versus 15% RSV, p < 0.0001; **5-8 years**: 26% versus <7% other viruses, p < 0.0001; **9-17 years**: 16% versus 9% influenza A, p = 0.007), and RSV was the most common detected in children ** < 1 year **(30% versus 23% picornavirus, p < 0.0001). Influenza A showed growing importance with age, and was the second most common virus detected in children >9 years old (9% influenza A versus <4% other viruses, p < 0.0001).

### Codetection of respiratory viruses

Out of the total of 10'629 samples analysed, 82 were positive for two viruses (Table 2). No sample was positive for more than two viruses. This corresponds to a codetection rate of 0.8% with DFA.

**Table 2:** Number of nasopharyngeal aspirates with viral codetections by DFA

	picorna	hMPV	PIV	IFB	IFA	RSV
**ADV**	9	2	4	0	2	10
**RSV**	29	0	0	1	7	
**IFA**	1	0	4			
**PIV**	9	1				
**hMPV**	3					

ADV = adenovirus; RSV = respiratory syncytial virus; IFA = influenza A; IFB = influenza B; PIV = parainfluenza viruses 1-3; hMPV = human metapneumovirus; picorna = respiratory picornaviruses

With the xTag Respiratory Viral Panel (Luminex) 10.2% of samples were positive for two viruses (in 81% of these a respiratory picornavirus was present). In 0.8% of samples we detected three respiratory viruses.

## Discussion

In order to determine the value of DFA in conducting epidemiological studies on respiratory viruses now that assays for respiratory picornaviruses and hMPV are available, we retrospectively analysed the results of 12 years of DFA screening of viral pathogens in hospitalized children with respiratory disease.

Respiratory picornaviruses were the most common viral pathogens detected overall in our study, with the exception of patients <1 year in whom RSV was detected more often, confirming the results of recent studies based on molecular methods [25,26]. PCR detection of respiratory picornaviruses suggests a previously unexpected role [27,28] in severe respiratory disease [25,26,29], but this issue is still debated, given that viral genome can also be detected by PCR many weeks after an acute viral infection [17], or even in entirely asymptomatic children [16,30]. DFA assays need a high viral load to score positive, so a positive result may be more indicative of an acute infection caused by the virus [18,19]. Our high detection rate of respiratory picornaviruses by DFA in hospitalized patients therefore supports their high burden of disease.

The introduction of the hMPV and respiratory picornavirus assays in 2006 increased the positivity rate of our DFA screening from 35% to 58%. For comparison, PCR methods in our laboratory and in the literature usually reach positivity rates of well over 70%, in large part due to a higher detection of respiratory picornaviruses [6,7,26,31]. DFA's lower sensitivity, in particular for respiratory picornaviruses, can however be seen as an advantage considering the difficulty in interpreting the clinical significance of PCR-positive results, as described above.

Another common issue in PCR-based studies is the high codetection rate, with on average about 20% of samples being positive for two or more viruses [3,26]. With the xTag Respiratory Viral Panel we detected more than one virus in 11% of samples. In 81% of these a respiratory picornavirus was present. The clinical significance of these "coinfections" remains unclear because of the high sensitivity of PCR [3,32,33], especially for respiratory picornaviruses. It is difficult to determine whether both or only one and which of the codetected pathogens is causing the acute illness [30]. Our lower rate of codetection by DFA suggests that most codetections detected by PCR may indicate consecutive infections.

With the exception of hMPV, our study confirms known patterns of seasonality and age distribution for the studied viruses [22,26,34,35]. It has been postulated that hMPV has a biennial "large-early" and "small-late" season cycle [26,36-38]. We observed an unexpectedly large epidemic in early 2010, which was observed simultaneously in many cities throughout Germany (personal communication, Prof. O. Adams, University of Düsseldorf). Given the recent discovery of hMPV [10], epidemiological studies so far have covered a short time interval and continued monitoring is necessary.

Currently, PCR is considered the most adapted technique to conduct epidemiological studies on respiratory viruses. In contrast to molecular methods, DFA is low in cost and has a rapid turnaround time [21]. Assays can be performed many times a day, and one does not have to wait for a certain number of samples to be collected to start a run. The samples can be screened for many different viruses simultaneously ("multiplex"). Results are usually available within 2-3 hours [39]. These aspects make DFA a method widely and often used in clinical routine, and this concurrently provides the data for ongoing, real-time surveillance of circulating viral pathogens on a large scale. Our systematic monitoring led for example to the early detection of the unexpectedly large hMPV epidemic mentioned above.

## Conclusions

In conclusion, DFA's clinical relevance, flexibility and capacity to conduct "multiplex" assays at very low cost make it a valuable diagnostic tool, and now that its range of viral detection has been broadened to include hMPV and especially respiratory picornaviruses, allows for long-term, systematic, real-time monitoring of local epidemiology in pediatric populations.

## Abbreviations

DFA: direct immunofluorescence assay; ADV: adenovirus; RSV: respiratory syncytial virus; IFA: influenza A; IFB: influenza B; PIV 1-3: parainfluenza viruses 1-3; hMPV: human metapneumovirus; picorna: respiratory picornaviruses;

## Competing interests

The authors declare that they have no competing interests.

## Authors' contributions

CDS participated in statistical analysis, data interpretation, drafting of the manuscript, and critical revision of the manuscript. CA participated in study design and sample acquisition. MGH participated in study design, data interpretation, and critical revision of the manuscript. KM participated in study design and critical revision of the manuscript. MTB participated in study design, data interpretation, drafting of the manuscript and critical revision of the manuscript. All authors read and approved the final manuscript.

## Pre-publication history

The pre-publication history for this paper can be accessed here:

http://www.biomedcentral.com/1471-2334/11/41/prepub

## References

[B1] Hon KL Nelson EA Gender disparity in paediatric hospital admissions Ann Acad Med Singapore 2006 35 12 882 888 17219000

[B2] Juven T Mertsola J Waris M Leinonen M Meurman O Roivainen M Eskola J Saikku P Ruuskanen O Etiology of community-acquired pneumonia in 254 hospitalized children Pediatr Infect Dis J 2000 19 4 293 298 10.1097/00006454-200004000-00006 10783017

[B3] Tregoning JS Schwarze J Respiratory viral infections in infants: causes, clinical symptoms, virology, and immunology Clin Microbiol Rev 2010 23 1 74 98 10.1128/CMR.00032-09 20065326 PMC2806659

[B4] Templeton KE Why diagnose respiratory viral infection? J Clin Virol 2007 40 Suppl 1 S2 4 10.1016/S1386-6532(07)70002-1 18162250 PMC7128291

[B5] Papadopoulos NG Bates PJ Bardin PG Papi A Leir SH Fraenkel DJ Meyer J Lackie PM Sanderson G Holgate ST Johnston SL Rhinoviruses infect the lower airways J Infect Dis 2000 181 6 1875 1884 10.1086/315513 10837165

[B6] Jacques J Bouscambert-Duchamp M Moret H Carquin J Brodard V Lina B Motte J Andreoletti L Association of respiratory picornaviruses with acute bronchiolitis in French infants J Clin Virol 2006 35 4 463 466 10.1016/j.jcv.2005.11.009 16406692

[B7] Papadopoulos NG Moustaki M Tsolia M Bossios A Astra E Prezerakou A Gourgiotis D Kafetzis D Association of rhinovirus infection with increased disease severity in acute bronchiolitis Am J Respir Crit Care Med 2002 165 9 1285 1289 10.1164/rccm.200112-118BC 11991880

[B8] Korppi M Kotaniemi-Syrjanen A Waris M Vainionpaa R Reijonen TM Rhinovirus-associated wheezing in infancy: comparison with respiratory syncytial virus bronchiolitis Pediatr Infect Dis J 2004 23 11 995 999 10.1097/01.inf.0000143642.72480.53 15545853

[B9] Johnston SL Pattemore PK Sanderson G Smith S Lampe F Josephs L Symington P O'Toole S Myint SH Tyrrell DA Holgate ST Community study of role of viral infections in exacerbations of asthma in 9-11 year old children Bmj 1995 310 6989 1225 1229 7767192 10.1136/bmj.310.6989.1225 PMC2549614

[B10] van den Hoogen BG de Jong JC Groen J Kuiken T de Groot R Fouchier RA Osterhaus AD A newly discovered human pneumovirus isolated from young children with respiratory tract disease Nat Med 2001 7 6 719 724 10.1038/89098 11385510 PMC7095854

[B11] Freymuth F Vabret A Legrand L Dina J Gouarin S Cuvillon-Nimal D Brouard J [Human metapneumovirus] Pathol Biol (Paris) 2009 57 2 133 141 18515017 10.1016/j.patbio.2008.04.005 PMC7126272

[B12] Manoha C Espinosa S Aho SL Huet F Pothier P Epidemiological and clinical features of hMPV, RSV and RVs infections in young children J Clin Virol 2007 38 3 221 226 10.1016/j.jcv.2006.12.005 17241812

[B13] Williams JV Harris PA Tollefson SJ Halburnt-Rush LL Pingsterhaus JM Edwards KM Wright PF Crowe JE Jr Human metapneumovirus and lower respiratory tract disease in otherwise healthy infants and children N Engl J Med 2004 350 5 443 450 10.1056/NEJMoa025472 14749452 PMC1831873

[B14] Thavagnanam S Christie SN Doherty GM Coyle PV Shields MD Heaney LG Respiratory viral infection in lower airways of asymptomatic children Acta Paediatr 2010 99 3 394 8 10.1111/j.1651-2227.2009.01627.x 20003105 PMC7159555

[B15] van der Zalm MM van Ewijk BE Wilbrink B Uiterwaal CS Wolfs TF van der Ent CK Respiratory pathogens in children with and without respiratory symptoms J Pediatr 2009 154 3 396 400 400 e391. 10.1016/j.jpeds.2008.08.036 18823911 PMC7094528

[B16] van Gageldonk-Lafeber AB Heijnen ML Bartelds AI Peters MF van der Plas SM Wilbrink B A case-control study of acute respiratory tract infection in general practice patients in The Netherlands Clin Infect Dis 2005 41 4 490 497 10.1086/431982 16028157 PMC7107976

[B17] Jartti T Lehtinen P Vuorinen T Koskenvuo M Ruuskanen O Persistence of rhinovirus and enterovirus RNA after acute respiratory illness in children J Med Virol 2004 72 4 695 699 10.1002/jmv.20027 14981776

[B18] Madeley CR Peiris JS Methods in virus diagnosis: immunofluorescence revisited J Clin Virol 2002 25 2 121 134 10.1016/S1386-6532(02)00039-2 12367646

[B19] Schindera C Kraemer AL Regamey N Aebi C Gorgievski-Hrisoho M Barbani MT Immunofluorescence versus xTAG multiplex PCR for the detection of respiratory picornavirus infections in children J Clin Virol 2010 48 3 223 225 10.1016/j.jcv.2010.04.005 20471907 PMC7172693

[B20] Fabbiani M Terrosi C Martorelli B Valentini M Bernini L Cellesi C Cusi MG Epidemiological and clinical study of viral respiratory tract infections in children from Italy J Med Virol 2009 81 4 750 756 10.1002/jmv.21457 19235872 PMC7167005

[B21] Freymuth F Vabret A Cuvillon-Nimal D Simon S Dina J Legrand L Gouarin S Petitjean J Eckart P Brouard J Comparison of multiplex PCR assays and conventional techniques for the diagnostic of respiratory virus infections in children admitted to hospital with an acute respiratory illness J Med Virol 2006 78 11 1498 1504 10.1002/jmv.20725 16998894 PMC7159369

[B22] Irmen KE Kelleher JJ Use of monoclonal antibodies for rapid diagnosis of respiratory viruses in a community hospital Clin Diagn Lab Immunol 2000 7 3 396 403 10799452 10.1128/cdli.7.3.396-403.2000 PMC95885

[B23] Tang LF Wang TL Tang HF Chen ZM Viral pathogens of acute lower respiratory tract infection in China Indian Pediatr 2008 45 12 971 975 19129564

[B24] Barbani MT Gorgievski-Hrisoho M Rapid detection of respiratory picornaviruses in nasopharyngeal aspirates by immunofluorescence assay J Clin Virol 2009 45 3 245 248 10.1016/j.jcv.2009.05.008 19502108 PMC7172351

[B25] Jartti T Lehtinen P Vuorinen T Osterback R van den Hoogen B Osterhaus AD Ruuskanen O Respiratory picornaviruses and respiratory syncytial virus as causative agents of acute expiratory wheezing in children Emerg Infect Dis 2004 10 6 1095 1101 15207063 10.3201/eid1006.030629 PMC3323183

[B26] Weigl JA Puppe W Meyer CU Berner R Forster J Schmitt HJ Zepp F Ten years' experience with year-round active surveillance of up to 19 respiratory pathogens in children Eur J Pediatr 2007 166 9 957 966 10.1007/s00431-007-0496-x 17569085 PMC7087302

[B27] Aymard M Chomel JJ Allard JP Thouvenot D Honegger D Floret D Boissel JP Collet JP Durr F Gillet J Bossard N Lyon L Epidemiology of viral infections and evaluation of the potential benefit of OM-85 BV on the virologic status of children attending day-care centers Respiration 1994 61 Suppl 1 24 31 10.1159/000196377 7800968 PMC7182644

[B28] Mackay IM Human rhinoviruses: the cold wars resume J Clin Virol 2008 42 4 297 320 10.1016/j.jcv.2008.04.002 18502684 PMC7108405

[B29] Louie JK Roy-Burman A Guardia-Labar L Boston EJ Kiang D Padilla T Yagi S Messenger S Petru AM Glaser CA Schnurr DP Rhinovirus associated with severe lower respiratory tract infections in children Pediatr Infect Dis J 2009 28 4 337 339 10.1097/INF.0b013e31818ffc1b 19258921

[B30] Nokso-Koivisto J Kinnari TJ Lindahl P Hovi T Pitkaranta A Human picornavirus and coronavirus RNA in nasopharynx of children without concurrent respiratory symptoms J Med Virol 2002 66 3 417 420 10.1002/jmv.2161 11793396 PMC7166414

[B31] Legg JP Warner JA Johnston SL Warner JO Frequency of detection of picornaviruses and seven other respiratory pathogens in infants Pediatr Infect Dis J 2005 24 7 611 616 10.1097/01.inf.0000168747.94999.aa 15999002

[B32] Aberle JH Aberle SW Pracher E Hutter HP Kundi M Popow-Kraupp T Single versus dual respiratory virus infections in hospitalized infants: impact on clinical course of disease and interferon-gamma response Pediatr Infect Dis J 2005 24 7 605 610 10.1097/01.inf.0000168741.59747.2d 15999001

[B33] Midulla F Scagnolari C Bonci E Pierangeli A Antonelli G De Angelis D Berardi R Moretti C Respiratory syncytial virus, human bocavirus and rhinovirus bronchiolitis in infants Arch Dis Child 2010 95 1 35 41 10.1136/adc.2008.153361 19822538

[B34] Duppenthaler A Gorgievski-Hrisoho M Frey U Aebi C Two-year periodicity of respiratory syncytial virus epidemics in Switzerland Infection 2003 31 2 75 80 10.1007/s15010-002-3124-8 12682811

[B35] Monto AS Epidemiology of influenza Vaccine 2008 26 Suppl 4 D45 48 10.1016/j.vaccine.2008.07.066 19230159

[B36] Aberle SW Aberle JH Sandhofer MJ Pracher E Popow-Kraupp T Biennial spring activity of human metapneumovirus in Austria Pediatr Infect Dis J 2008 27 12 1065 1068 10.1097/INF.0b013e31817ef4fd 18978517

[B37] Heininger U Kruker AT Bonhoeffer J Schaad UB Human metapneumovirus infections--biannual epidemics and clinical findings in children in the region of Basel, Switzerland Eur J Pediatr 2009 168 12 1455 1460 10.1007/s00431-009-0949-5 19238433

[B38] Rafiefard F Yun Z Orvell C Epidemiologic characteristics and seasonal distribution of human metapneumovirus infections in five epidemic seasons in Stockholm, Sweden, 2002-2006 J Med Virol 2008 80 9 1631 1638 10.1002/jmv.21242 18649344

[B39] Landry ML Cohen S Ferguson D Prospective study of human metapneumovirus detection in clinical samples by use of light diagnostics direct immunofluorescence reagent and real-time PCR J Clin Microbiol 2008 46 3 1098 1100 10.1128/JCM.01926-07 18184854 PMC2268359

